# Operational daily evapotranspiration mapping at field scale based on SSEBop model and spatiotemporal fusion of multi-source remote sensing data

**DOI:** 10.1371/journal.pone.0264133

**Published:** 2022-02-17

**Authors:** Qifeng Zhuang, Hua Shao, Dongliang Guan

**Affiliations:** College of Geomatics Science and Technology, Nanjing Tech University, Nanjing, China; Soil and Water Resources Institute ELGO-DIMITRA, GREECE

## Abstract

Accurate understanding of daily evapotranspiration (ET) at field scale is of great significance for agricultural water resources management. The operational simplified surface energy balance (SSEBop) model has been applied to estimate field scale ET with Landsat satellite imagery. However, there is still uncertainty in the ET time reconstruction for cloudy days based on limited clear days’ Landsat ET fraction (*ET*_*f*_) computed by SSEBop. The Moderate Resolution Imaging Spectroradiometer (MODIS) remote sensing data can provide daily surface observation over clear-sky areas. This paper presented an enhanced gap-filling scheme for the SSEBop ET model, which improved the temporal resolution of Landsat *ET*_*f*_ through the spatio-temporal fusion with SSEBop MODIS *ET*_*f*_ on clear days and increased the time reconstruction accuracy of field-scale ET. The results were validated with the eddy covariance (EC) measurements over cropland in northwestern China. It indicated that the improved scheme performed better than the original SSEBop Landsat approach in daily ET estimation, with higher Nash-Sutcliffe efficiency (NSE, 0.75 vs. 0.70), lower root mean square error (RMSE, 0.95 mm·d^-1^ vs. 1.05 mm·d^-1^), and percent bias (PBias, 16.5% vs. 25.0%). This fusion method reduced the proportion of deviation (13.3% vs. 25.5%) in the total errors and made the random error the main proportion, which can be reduced over time and space in regional ET estimation. It also evidently improved the underestimation of crop ET by the SSEBop Landsat scheme during irrigation before sowing and could more accurately describe the synergistic changes of soil moisture and cropland ET. The proposed MODIS and Landsat *ET*_*f*_ fusion can significantly improve the accuracy of SSEBop in estimating field-scale ET.

## 1. Introduction

Evapotranspiration (ET) is the loss of water through soil evaporation and plant transpiration, and it connects the global water and energy cycles. Understanding the cropland water use in agriculture water management requires detailed information about field-scale daily crop water consumption. This need becomes particularly relevant in areas characterized by increasing limitations in water resources [1], such as in western China.

ET can be observed by ground site measurements or estimated by satellite-driven models proposed in recent decades. The site observation methods, e.g., the large aperture scintillometer (LAS) and eddy covariance (EC) systems, can obtain the sensible and latent heat flux in the source area, which represents the water and heat transfer intensity within hundreds of meters [2]. The remote sensing ET models can be applied for cropland irrigation management at the regional scale. Over the last few decades, several remote sensing approaches have been developed to estimate ET on different spatio-temporal scales. Mu et al. [3] introduced the MOD16 ET technique, which used the well-known Penman-Monteith (PM) equation and MODIS (Moderate Resolution Imaging Spectroradiometer) remote sensing data to compute evaporation from the soil and moist canopy, as well as plant transpiration. Fisher et al. [4] developed a satellite vegetation index-based ET model that employed the PM and Priestley-Taylor (PT) equations to calculate total ET from canopy transpiration, soil evaporation, and interception evaporation. The surface energy balance (SEB) models solved the net radiation (*R*_*n*_), the soil heat flux (*G*), and the sensible heat flux (*H*) to derive the latent heat flux (*LE*) as the reminder. These models exploit the land surface temperature (LST) collected from remote sensing to indicate surface water status. Some of these models, such as the Two-Source Energy Balance (TSEB) [5] and Atmosphere-Land Exchange Inverse (ALEXI) [6], are more physically based and explicitly simulate the soil vegetation-atmosphere exchange processes. Others, such as the Surface Energy Balance Algorithm for Land (SEBAL) [7], Mapping Evapotranspiration at High Resolution and with Internalized Calibration (METRIC) [8], and the Operational Simplified Surface Energy Balance Algorithm (SSEBop) [9], have been developed to reduce required model inputs by using semi-empirical approaches or within-scene scaling.

There have been several inter-comparisons of these models in various contexts, with uncertainty in the ET estimates from different models ranging from 5 to 50% [10, 11]. The land surface temperature offers essential information on the moisture status of the surface and subsoil, which is required for calculating ET and predicting the onset and severity of droughts [12]. The LST-based surface energy balance models have more potential to indicate the changes of ET caused by soil water changes. The Priestley-Taylor or Penman-Monteith equation-based models require soil moisture as input to accurately reflect ET change [13]. However, due to the complexity of aerodynamic parameters (i.e., the aerodynamic roughness and aerodynamic temperature for heat transfer) of the underlying surface [14, 15], these models may produce errors in calculating sensible heat flux, resulting in erroneous simulated ET.

The SSEBop is a simplified surface energy balance model for regional ET estimates. It directly determines latent heat flux without resolving all surface energy balance components, such as the sensible heat. This is useful for scientific studies of hydrological processes, water management authorities, and farmers making water budgeting or irrigation planning decisions [16]. The SSEBop model has been applied to calculating global ET based on the MODIS and reanalysis data. To obtain finer resolution ET using Landsat satellite data, Senay et al. [16] turned to inspect a scene constrained limiting ratio between overpass Landsat ET fraction (*ET*_*f*_) and its nearest MODIS *ET*_*f*_ to fill the voids of the 9 to 15 Landsat images in a year. The daily ET values for dates between overpass images were derived by the daily potential ET and its nearest respective overpass *ET*_*f*_.

However, the limited overpass Landsat image can not represent the specific change of crop water consumption over time. For example, if there is a lack of satellite overpass thermal infrared data, ET estimates may be dubious when irrigation occurs. Recent research has applied the Landsat-MODIS reflectance, LST, and ET data fusion methodologies to produce high spatio-temporal resolution ET products using the SEBS or DisAlexi models [1, 17–20]. Bei et al. [18] found that the fusion of Landsat-MODIS vegetation index yielded higher estimation accuracy of ET than the fusion of reflectance using the Priestley-Taylor model. This is because more fusion times may amplify errors from remote sensing data and fusion algorithms. There is reduced error propagation because of the fusion of a single vegetation index band.

This study aims to show how well a Landsat-MODIS data fusion framework can improve the SSEBop estimated actual daily ET at the field scale. This methodology initially used the MODIS and Landsat images to estimate actual ET under clear-sky conditions. Second, the ET fraction was selected as the critical parameter for data fusion by the Spatial and Temporal Adaptive Reflectance Fusion Model (STARFM) [21]. Finally, the results were assessed by the automatic weather systems (AWS) and eddy covariance (EC) measurements over cropland in northwestern China and compared with the SSEBop estimates using only Landsat data. The analysis of the results was also listed in the discussion session.

## 2. Materials and methods

### 2.1. Study area and data

**Fig 1:**
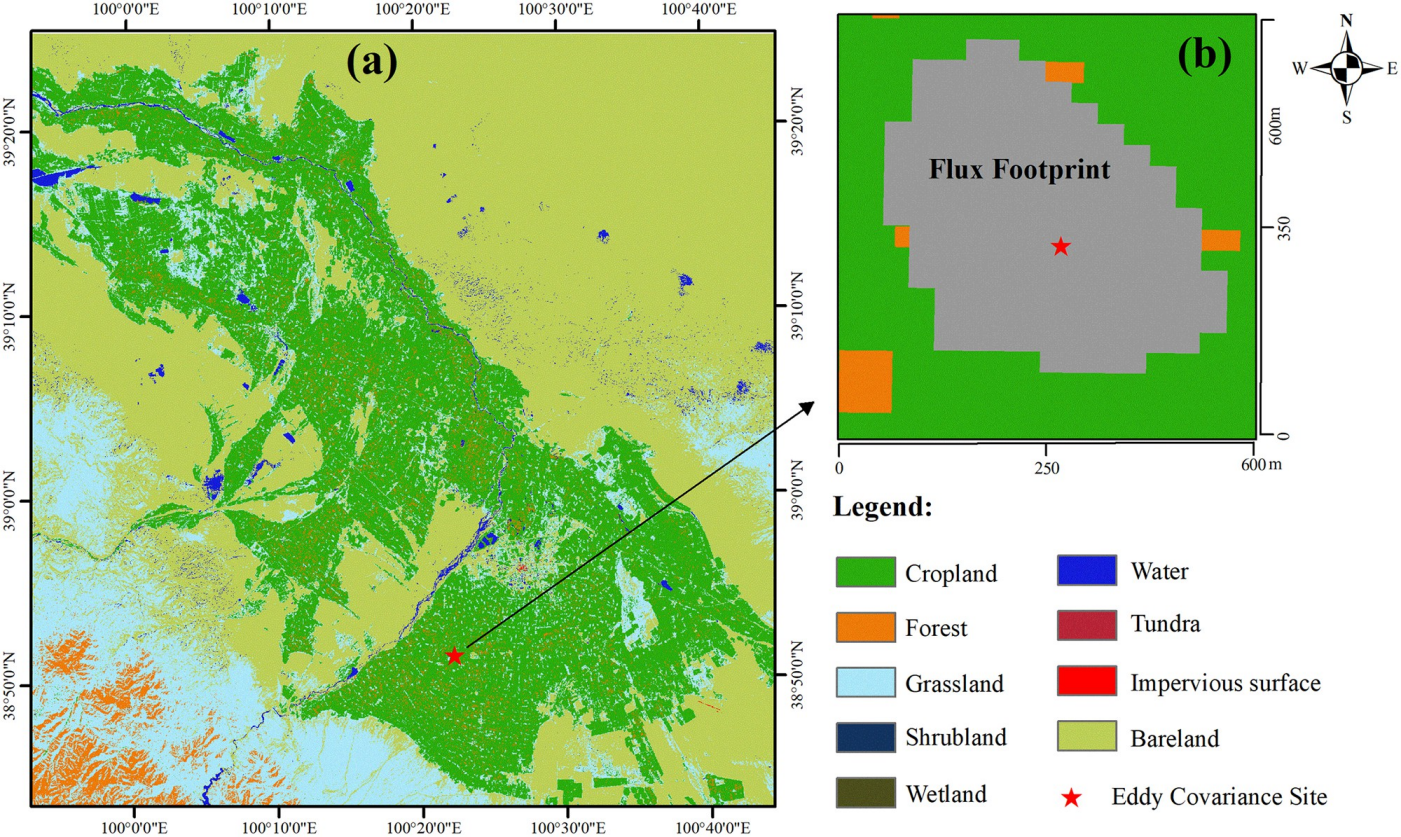
Land cover of the research area (a) and the EC station with its footprint climatology (b).

The Heihe River Basin (HRB) is the second-largest inland river basin in northwest China’s arid region, with the yearly precipitation ranging from 100 to 250 mm. The research area is located in an oasis-desert zone in the HRB’s middle reaches, with a range of around 200 km×200 km. Maize is the principal crop of the irrigated fields, which grows from April to October and consumes a big part of the HRB’s scarce water resources. From November to March, there is no crop in the fields. Fig 1 shows the land-use type in 2015 [22]; this region is mainly covered by croplands, grasslands, barren areas, etc. The grasslands area is close to the upper reaches of HRB.

The eddy covariance (EC) and automatic weather systems were installed through the Heihe Watershed Allied Telemetry Experimental Research (HiWATER) projects [23, 24]. The released EC products provide the half-hourly sensible heat flux (H) and latent heat flux (*LE*). The automatic weather system observations include the surface net radiation (*Rn*), the soil heat flux (*G*), soil temperature, soil moisture, wind velocity, precipitation, air temperature, humidity, etc. We used the Averaging Soil Thermocouple Probe (TCAV) method to correct the soil heat flow plate measured *G* [25]. We solved the energy non-closure and filled the *LE* gaps by the Bowen ratio correction and mean diurnal variation methods [11]. Further, we used the Flux Footprint Prediction (FFP) model and time series EC data to calculate the footprint climatology as the source area [2]. Fig 1 also shows the source area for the EC tower, and the ET pixels within this area were averaged for validation.

This research’s coarse remote sensing data include MODIS land surface temperature, reflectance, and albedo products from the Terra satellite with a resolution from 500m to 1000m. We unified the resolution of MODIS products to 500m through bilinear interpolation. The Landsat 8 Collection 2 Level 2 Science Product (L2SP) provides the finer surface temperature and reflectance with a resolution of 30m. The Google Earth Engine acquired ERA5 daily averages reanalysis data was adopted as regional meteorological inputs. It includes maximum and minimum air temperature, mean air temperature, wind speed, dew point temperature, and surface pressure.

### 2.2. Model description

The SSEBop model suggests that available net radiation primarily drives the surface energy balance process. Differences in LST can quantify a decline in ET due to water stress and other factors. SSEBop estimates actual evapotranspiration (*ET*_*a*_) by multiplying the ET fraction (*ET*_*f*_) and reference ET (*ET*_*r*_) as follows:

$$ET_{a}=ET_{f}\times k_{\text{max}}\times ET_{r}$$ (1)

where the *k*_*max*_ is the coefficient that scales the grass reference ET (*ET*_*r*_) into the level of a maximum ET experienced by an aerodynamically rougher crop, and a recommended value for *k*_*max*_ is 1.2. The SSEBop developed a new modification to estimate the solar radiation by assuming the “average-sky” condition and the daily net radiation (Rn24) can be computed from ERA5 minimum and maximum air temperature. The advantage of this method is that it does not need the observation of sunshine hours. The *ET*_*r*_ is calculated using the FAO-56 recommended PM equation with the ERA5 meteorological parameters and the Rn24. The *ET*_*f*_ is computed by the pixel-based land surface temperature *T*_*s*_, the estimated temperature *T*_*c*_ at the idealized “cold-wet” limit, and the predefined temperature difference *dT* between the”hot-dry” and”wet-cold” surfaces for each pixel as Eq (2):

$$ET_{f}=1-\frac{T_{s}-T_{c}}{dT}$$ (2)

**Fig 2:**
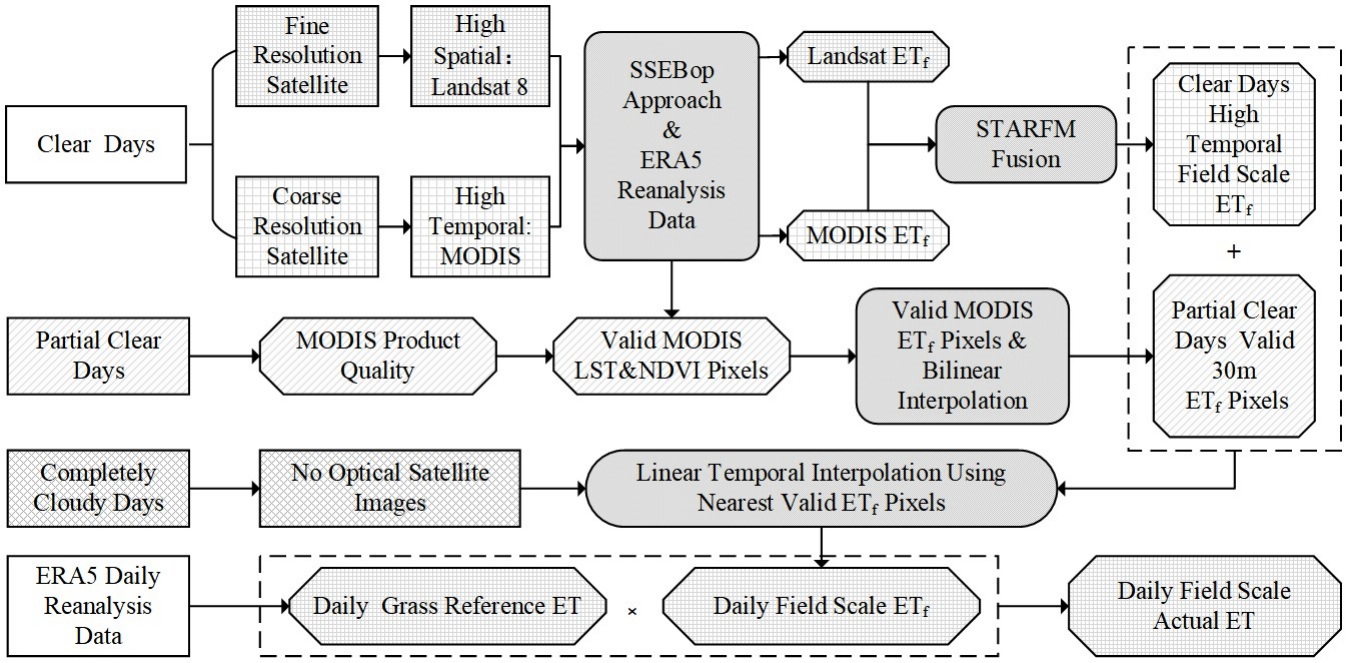
Flowchart of the enhanced approach for generating daily field-scale ET using SSEBop by MODIS and Landsat data.

For a more detailed introduction to the SSEBop model, please refer to the original literature [9, 12, 16, 26, 27]. The SSEBop model uses 9 to 15 Landsat images every year to simulate annual daily evapotranspiration (ET) at the field scale. The daily ET values for dates between overpass images are derived by the daily ET and its nearest respective overpass *ET*_*f*_. The lack of high-resolution images will lead to errors in *ET*_*f*_ simulation for many days. In this research, we used the MODIS and Landsat data to calculate the daily MODIS *ET*_*f*_ and Landsat *ET*_*f*_ on the overpass days and then applied the STARFM data fusion method to expand the time of Landsat like *ET*_*f*_ for clear-sky images. Because the fusion process needs to consider the values of surrounding pixels, for partial clear sky images, we used the bilinear interpolation method to resample MODIS *ET*_*f*_. This operation enables the valid *ET*_*f*_ pixel values to be as many as possible to ensure that the ET estimation error caused by data loss can be more avoided in the time reconstruction process. Fig 2 shows the overview of the enhanced SSEBop model methodology for estimating daily ET at the field scale. The results generated only by MODIS or Landsat are referred to as “SSEBop MODIS ET” and “SSEBop Landsat ET” after this, respectively. The results estimated by the fusion of MODIS and Landsat *ET*_*f*_ are called “SSEBop MODIS-Landsat Fusioned ET” in the following.

### 2.3. Evaluation method

We chose the correlation coefficient(r), coefficient of determination (R^2^), root mean square error (RMSE), Nash-Sutcliffe efficiency (NSE), and percent bias (PBias) statistics to evaluate the model results. The correlation coefficient (r) and coefficient of determination (R^2^) describe the degree of collinearity between simulated and measured data. The RMSE indicates error in the units of the constituent of interest. The NSE is a normalized statistic that determines the degree of residual variance vs. recorded data variance. The NSE value indicates how closely the observed vs. simulated data graphic fits the 1:1 line [28]. NSE is computed as shown in Eq (3):

$$\text{NSE}=1-\frac{\sum {\left(O-M\right)}^{2}}{\sum {\left(O-O^{mean}\right)}^{2}}$$ (3)

where *M* is the modeled ET data point, *O* is the EC observed ET, and *O*^*mean*^ is the mean of observed data for the evaluated constituent. Percent bias (PBias) measures the average tendency of the simulated data to be larger or smaller than their observed counterparts and is computed as:

$$\text{PBias}=\frac{\sum \left(M-O)\cdot (100\%\right)}{\sum O}$$ (4)

It is essential to identify the sources of bias and remove or reduce the bias primarily when the model is used in water balance or crop water-consuming monitoring. The separation of the error into bias and random components provides further helpful information for evaluating the ET models [26]. Obviously, for the same RMSE, a model with no bias is superior to a model with a bias since the random errors that dominate the RMSE will tend to compensate over time or space, thus conserving volumetric estimates. The mean bias error and random error were investigated separately for irrigated fields in the research area:

$$\text{MBE}=\frac{\sum \left(M-O\right)}{n}$$ (5)

$$\text{MSE}={\frac{\sum \left(M-O\right)}{n}}^{2}$$ (6)

$$\text{MSEe}=\text{MSE}-{(\text{MBE)}}^{\text{2}}$$ (7)

where MBE (mm) is the mean bias error, *n* is the number of paired data points. MSE is the mean squared error (mm^*2*^), RMSE (mm) is calculated as the square root of MSE. The calculation of the square of the random error (MSEe, mm^*2*^) as the difference between MSE and (MBE)^*2*^ can be made by rearranging the Eq (7). MSEe is the mean square error of the random error term “e”. The MSEe shows the variability of the error itself from the average error. Since the square error terms are additive, the relative contribution of the random error and the square of the MBE can be expressed as a percentage of the MSE, which is the total square error between the modeled and observed values.

## 3. Results

### 3.1. Daily net radiation

**Fig 3:**
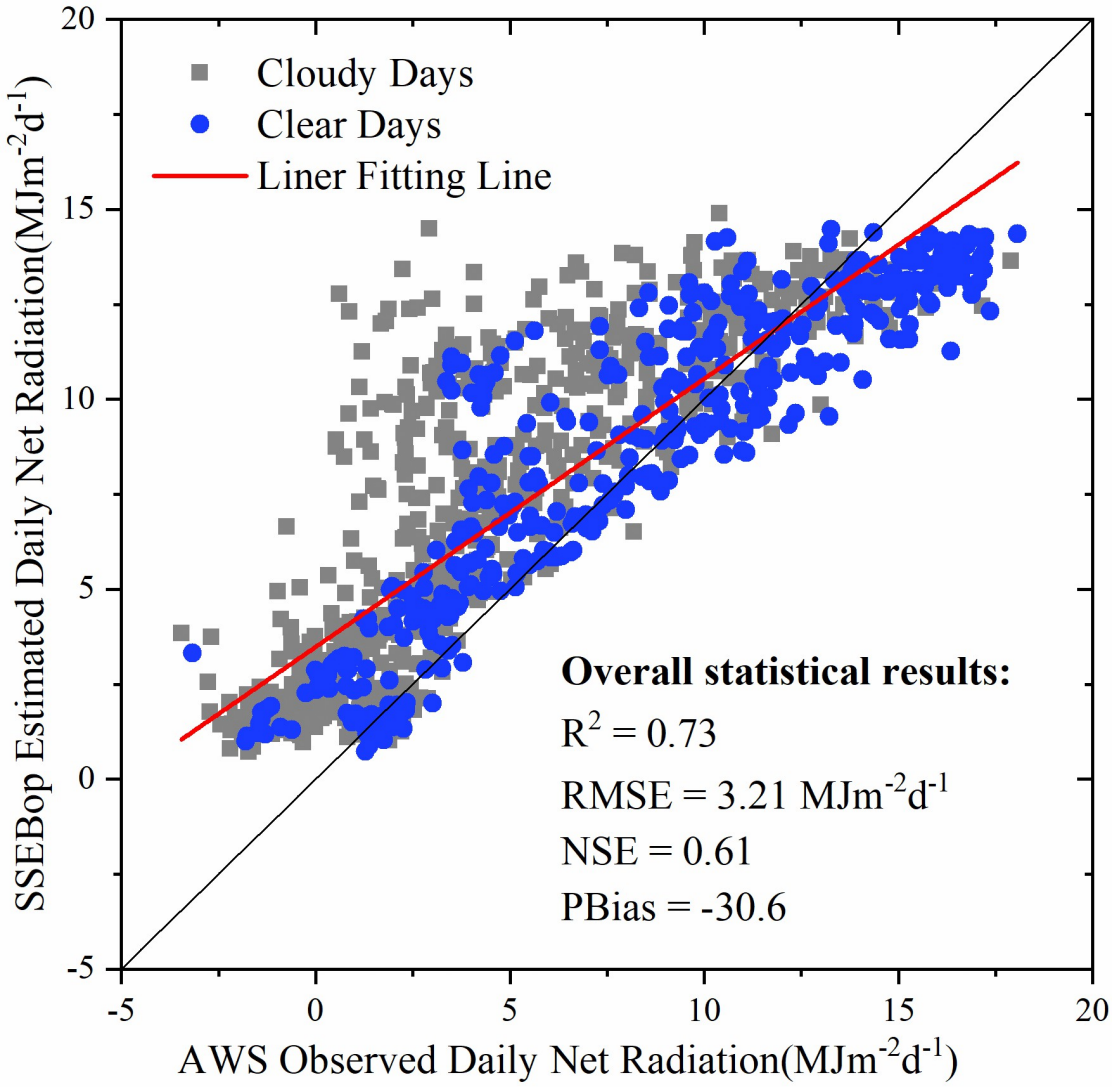
The scatter plot of the SSEBop “averaged-sky” net radiation versus the AWS observation from 2013 to 2015.

Fig 3 shows the daily net radiation results under the assumption of average clear sky estimated by the SSEBop model using ERA5 data. The model performance was regarded as acceptable if the NSE and the decisive coefficient (R^2^) were greater than 0.5 and 0.6, according to Santhi et al. (2001) [29], respectively. Therefore, the estimated daily net radiation had relatively high accuracy. The R^2^ was 0.73, the root mean square error (RMSE) was 3.21 MJm^-2^d^-1^, and the NSE was 0.61. The negative PBias indicated that the assumption of “averaged-sky” slightly overestimated the daily net radiation. The negative PBias of daily net radiation may cause higher *dT* and higher *ET*_*f*_ in Eq (2). According to Senay et al. [27], use of observed net radiation will improve the results; however, to establish the hot/dry boundary limit, the average-sky condition suffices for operational applications and its advantage for computational simplicity.

As shown by the grey dots in Fig 3, the overestimation of net radiation was mainly concentrated on cloudy days. The downward shortwave radiation in cloudy days is more affected by clouds, and the estimation method based on “average-sky” conditions may be insufficient. Since this research mainly evaluated the improvement of the time reconstruction method on the SSEBop model, we still adopted the original net radiation calculation method proposed in SSEBop.

### 3.2. Clear days’ SSEBop Landsat ET

**Fig 4:**
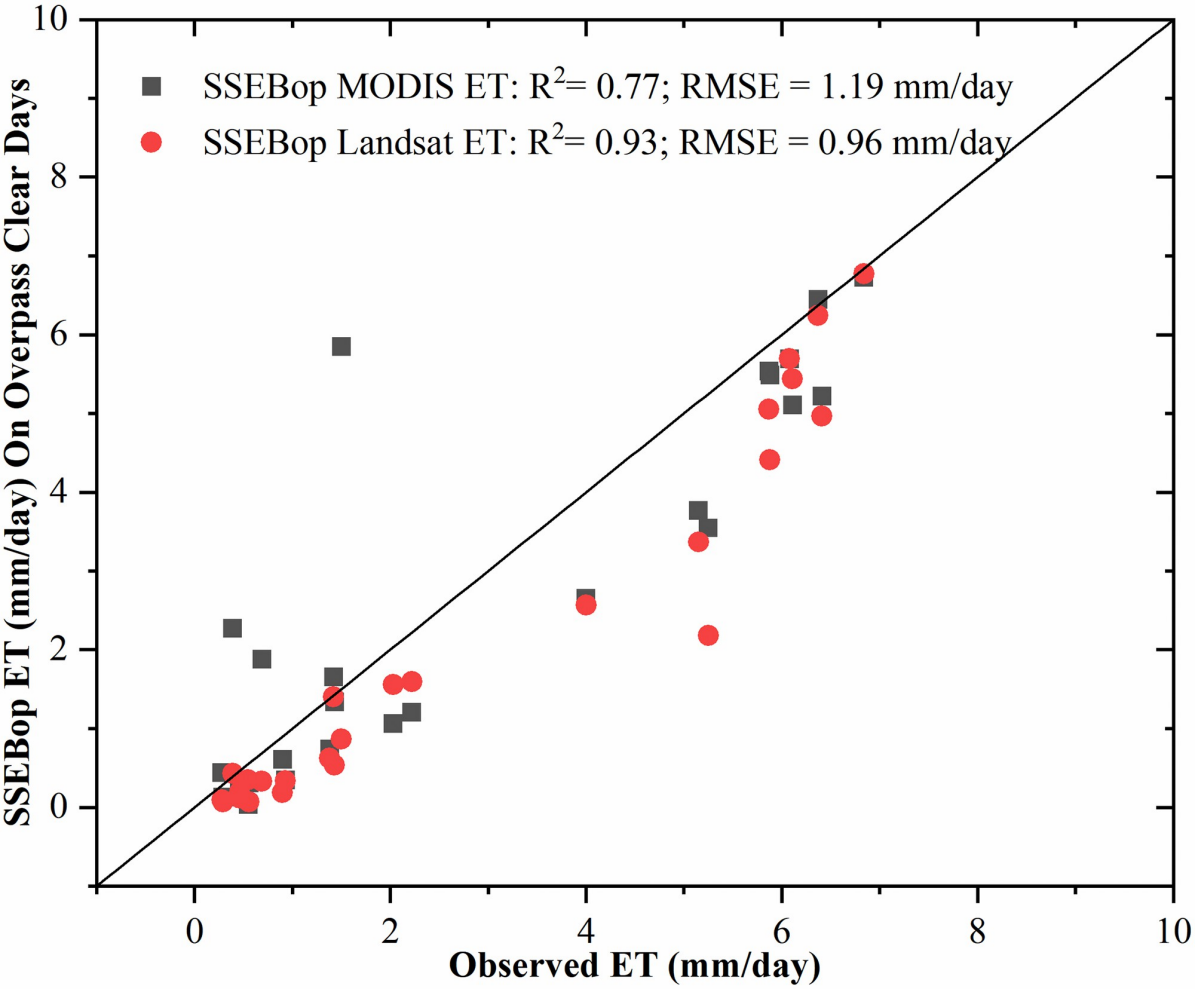
The validation of the estimated SSEBop ET using MODIS and Landsat data on the 26 Landsat 8 overpass clear sky days from 2013 to 2015.

We collected Landsat 8 overpass images on clear days covering the study area. There were 7, 11, and 8 images from 2013 to 2015, respectively. Fig 4 shows the scatter plot of the estimated SSEBop ET using MODIS and Landsat data on the 26 overpass clear sky days. It can be found from the Fig 4 that SSEBop MODIS ET on most clear days was consistent with the SSEBop Landsat ET. However, SSEBop Landsat ET generally had better estimation accuracy, with a higher coefficient of determination (R^2^) and lower root mean square error (RMSE) on clear days. The difference between the two results only lies in remote sensing data, which showed that it was more reasonable and feasible to use high-resolution Landsat data to estimate field-scale ET. It was also found that both the SSEBop MODIS and Landsat underestimated the actual ET. There may be two sources of this systematic error. One is the selection of the *k*_*max*_ parameter. The variation of *k*_*max*_ parameter usually ranges from 1 to 1.3. Local optimization can effectively improve the results. On the one hand, it may be the potential ET error caused by the uncertainty of ERA5 meteorological data. However, the primary purpose of this paper is to analyze whether the fusion of MODIS and Landsat can make the SSEBop model better estimate the daily field-scale ET.

### 3.3. Evaluation of daily ET time series

**Fig 5:**
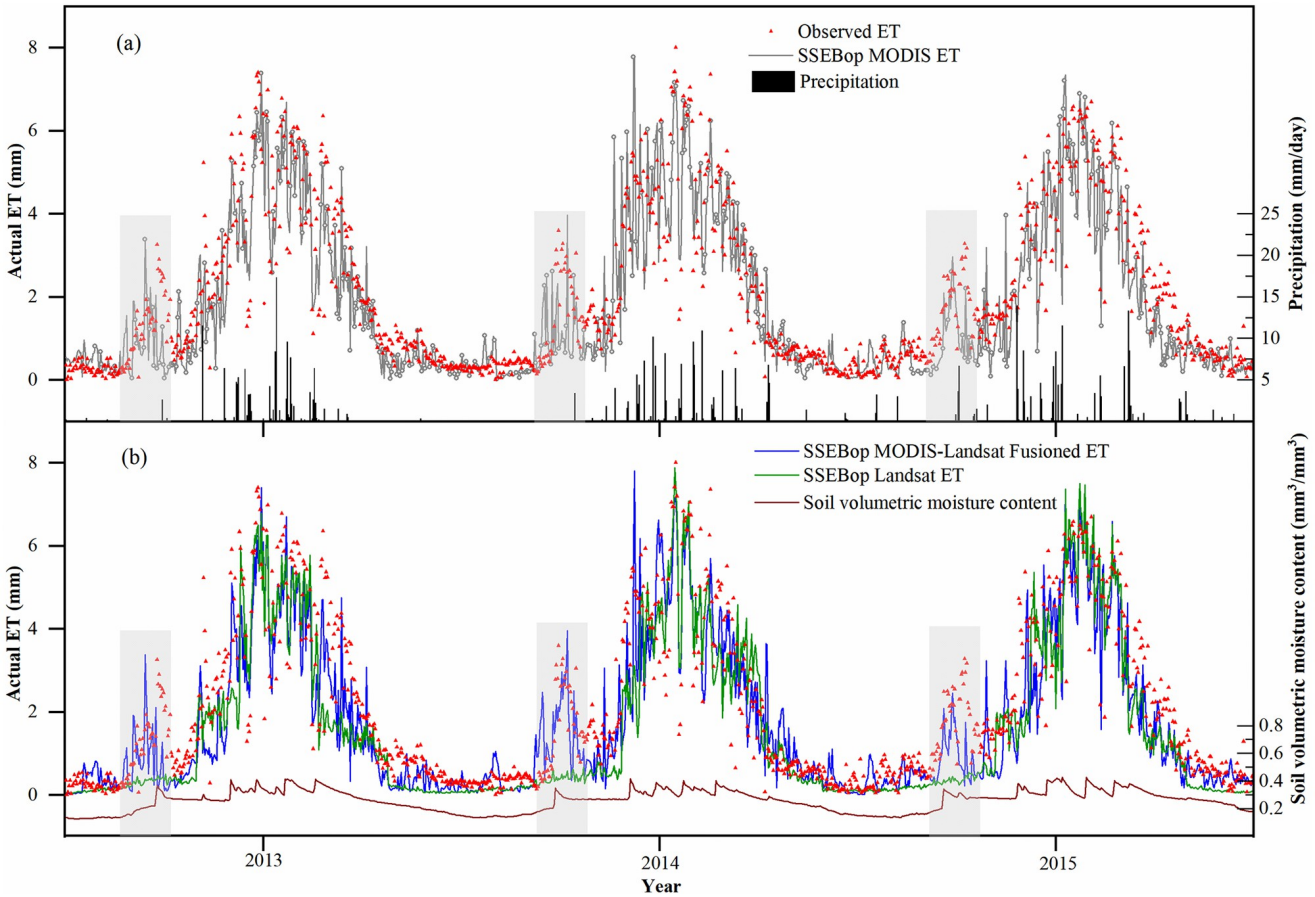
Comparisons of the SSEBop MODIS ET, SSEBop MODIS-Landsat Fusioned ET, SSEBop Landsat ET with the observed ET, soil moisture, and the precipitation.

Fig 5 compares the different methods estimated ET with the EC observation, soil moisture, and precipitation. In general, the three ways can well reflect the changing trends of ET in different phenological periods of crops. From April to September, the ET of crops from sowing to harvest showed a trend of gradually increasing and then decreasing progressively, roughly within 2-8mm. In July and August, the daily ET reached 6-8mm in the peak season of crop growth. The precipitation in this area mainly occurred from June to August, and the daily rainfall changed within 0-15mm. The soil volumetric water content varied from 0.2 to 0.4 throughout the crop growing season.

It can be seen from the shadow of Fig 5 that there was a sudden peak of ET in March every year, and the precipitation was almost zero at this time. By further comparing the soil moisture, the soil moisture also showed the same increasing trend. Therefore, we can conclude that this was the irrigation time in spring before sowing. Although there was no crop growth in the field, irrigation in spring can suddenly increase the ET from nearly 0 to 4 mm/day and gradually decrease after sowing. The SSEBop MODIS ET and SSEBop MODIS-Landsat Fusioned ET can effectively reflect this change. At the same time, SSEBop Landsat ET is not ideal, which underestimated the increase of ET caused by irrigation.

At this time, only considering NDVI is not enough. The surface temperature in the SSEBop model can effectively reflect the surface water status. The increase of soil moisture reduces the surface temperature, which increases *ET*_*f*_ and the estimated ET. However, it requires the input of surface temperature at this critical time. The SSEBop Landsat only uses limited Landsat images on clear days, so it can not guarantee to obtain the change of land surface temperature information. It also proves that the method in this study can improve the simulation accuracy of the ET time scale.

Table 1 shows the overall performance statistics of daily and monthly ET estimations using different methods. The NSE value close to 1 represents the overall high simulation accuracy of the model. All methods showed high r and R^2^, indicating the effectiveness of the SSEBop model with MODIS, Landsat, and ERA5 data in this area. The SSEBop MODIS had the best estimation accuracy. We found that this enhanced method (SSEBop MODIS-Landsat Fusioned ET) performed better than the original SSEBop Landsat approach, with higher NSE (0.75 vs. 0.70), lower RMSE (0.95 mmd^-1^ vs. 1.05 mmd^-1^), and PBias (16.5% vs. 25.0%) at daily scale and the monthly statistical results had a similar performance.

**Table 1:** The overall performance statistics of daily and monthly ET estimations using different methods.

	Methods	R^2^	NSE	RMSE	PBias
**Daily ET**	SSEBop MODIS	0.80	0.77	0.92	13.23
	SSEBop MODIS-Landsat Fusioned	0.79	0.75	0.95	16.51
	SSEBop Landsat	0.79	0.70	1.05	25.00
**Monthly ET**	SSEBop MODIS	0.96	0.94	13.19	13.23
	SSEBop MODIS-Landsat Fusioned	0.95	0.91	15.72	16.51
	SSEBop Landsat	0.93	0.83	21.69	25.00

**Fig 6:**
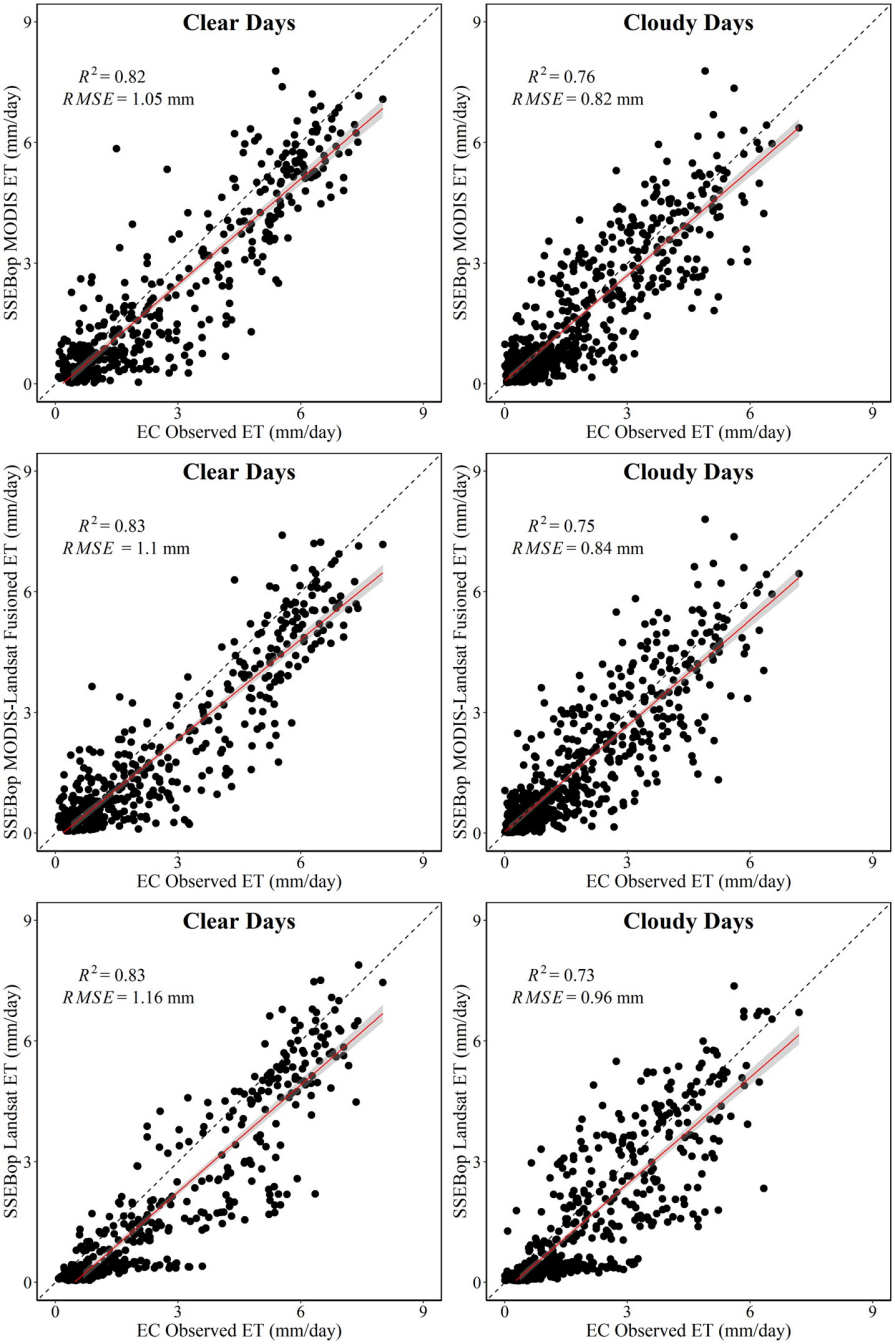
Comparison of estimated ET results with EC observations on clear and cloudy days.

Fig 6 shows the scatter plots for the three methods’ ET estimation versus EC observations. Generally, the clear days’ results had a higher coefficient of determination (R^2^) than cloudy days. However, there was a lower RMSE on cloudy days because the reduced net radiation made the crop have higher canopy resistance, and the ET declined. From 2013 to 2015, the average daily ET on clear days observed by EC was 2.7 mm/day, while cloudy days was 1.7 mm/day. Through comparison, it was found that the SSEBop MODIS-Landsat Fusioned ET had better performance on clear and cloudy days than the SSEBop Landsat ET.

Table 2 shows the error statics for the ET from the three methods. The relative contribution of the random error MSEe and the square of the MBE can be stated as a percentage of the MSE, which is the total square error between the modeled and observed values. The numbers in brackets in Table 2 represent the percentage of the corresponding error in the total error. From the statistical results, the MSE of SSEBop MODIS ET was the smallest, followed by SSEBop MODIS-Landsat Fusioned ET, and SSEBop Landsat ET was the largest. The percentage of random error in the total error of the three methods also had the same variation law, which was 90.6%, 86.7%, and 74.5%, respectively. A method with no bias (MBE^2^) is better than a model with a bias for the same RMSE (MSE) since the random errors (MSEe) that dominate the RMSE (MSE) will tend to compensate over time or space. Therefore, we can conclude that the fusion of MODIS and Landsat *ET*_*f*_ can effectively reduce the total error of field-scale daily ET estimation and increase the proportion of random error. In this way, random error reduction will be more evident than that of the original SSEBop Landsat ET in estimating long-time series ET, and the error accumulation phenomenon will be significantly improved.

**Table 2:** The error statics for the ET estimated by the three methods.

Errors	SSEBop MODIS ET	SSEBop MODIS-Landsat Fusioned ET	SSEBop Landsat ET
MSE	0.85	0.90	1.10
MBE^2^(%)	0.08(9.4)	0.12(13.3)	0.28(25.5)
MSEe(%)	0.77(90.6)	0.78(86.7)	0.82(74.5)

### 3.4. ET spatial patterns

**Fig 7:**
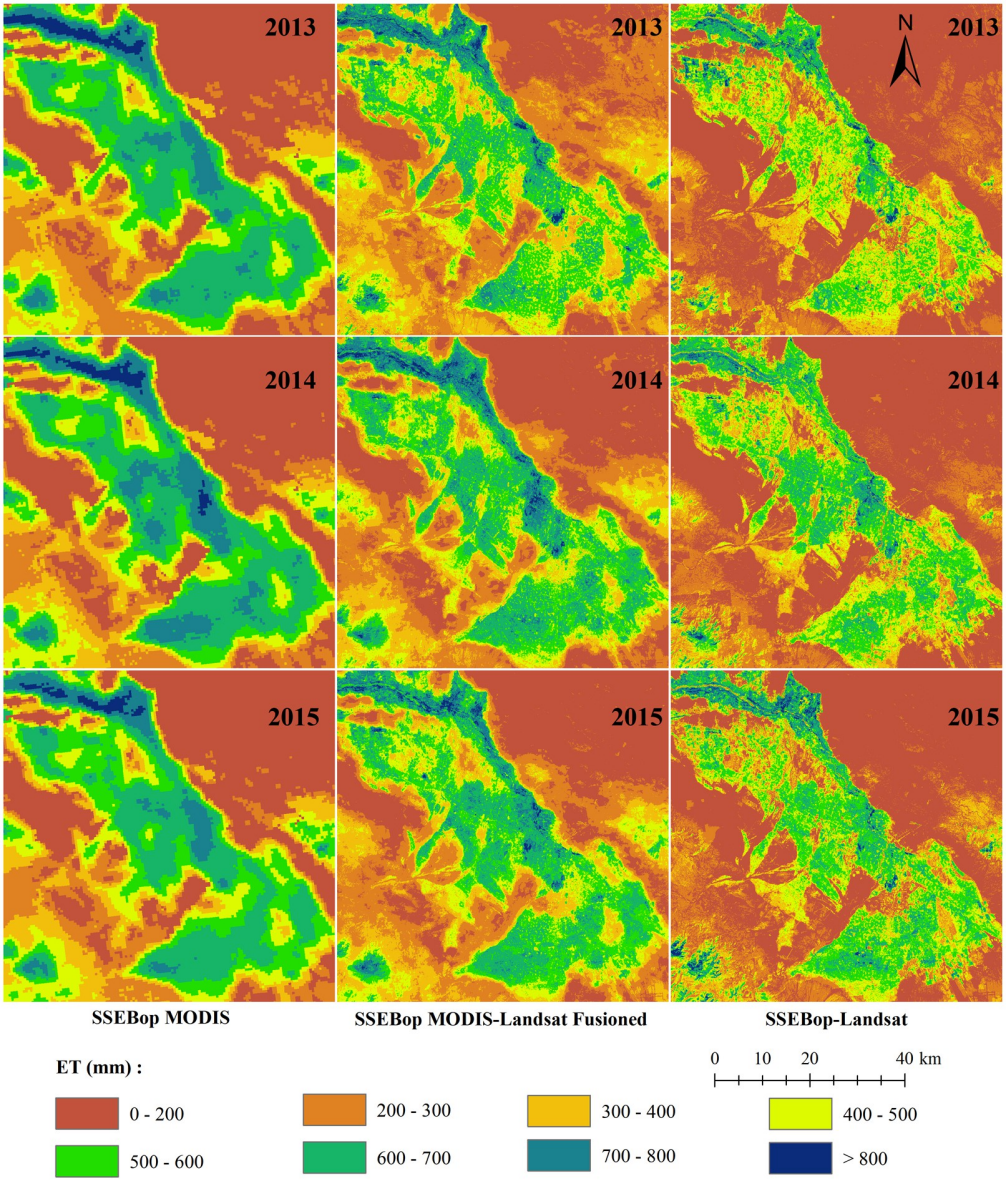
Yearly ET maps generated by the SSEBop MODIS, SSEBop MODIS-Landsat Fusioned, and SSEBop Landsat.

The validation and comparison of the previous chapters were carried out at the site scale, and the comparison of different methods in the region is equally important. Fig 7 shows the spatial distribution of annual evapotranspiration calculated by three methods. The same ET scale showed that the spatial distribution of SSEBop-MODIS and SSEBop MODIS-Landsat Fusioned ET was closer, significantly higher than that estimated by SSEBop-Landsat. The annual ET showed that the cropland ET was considerably higher than the bare soil area. The area with the highest ET value was concentrated in the range of the Heihe River, which indicated that the SSEBop model could estimate the ET of different underlying surfaces. Through *ET*_*f*_ fusion, the SSEBop MODIS-Landsat Fusioned ET was closer to that of SSEBop MODIS in site validation and on the regional scale.

**Fig 8:**
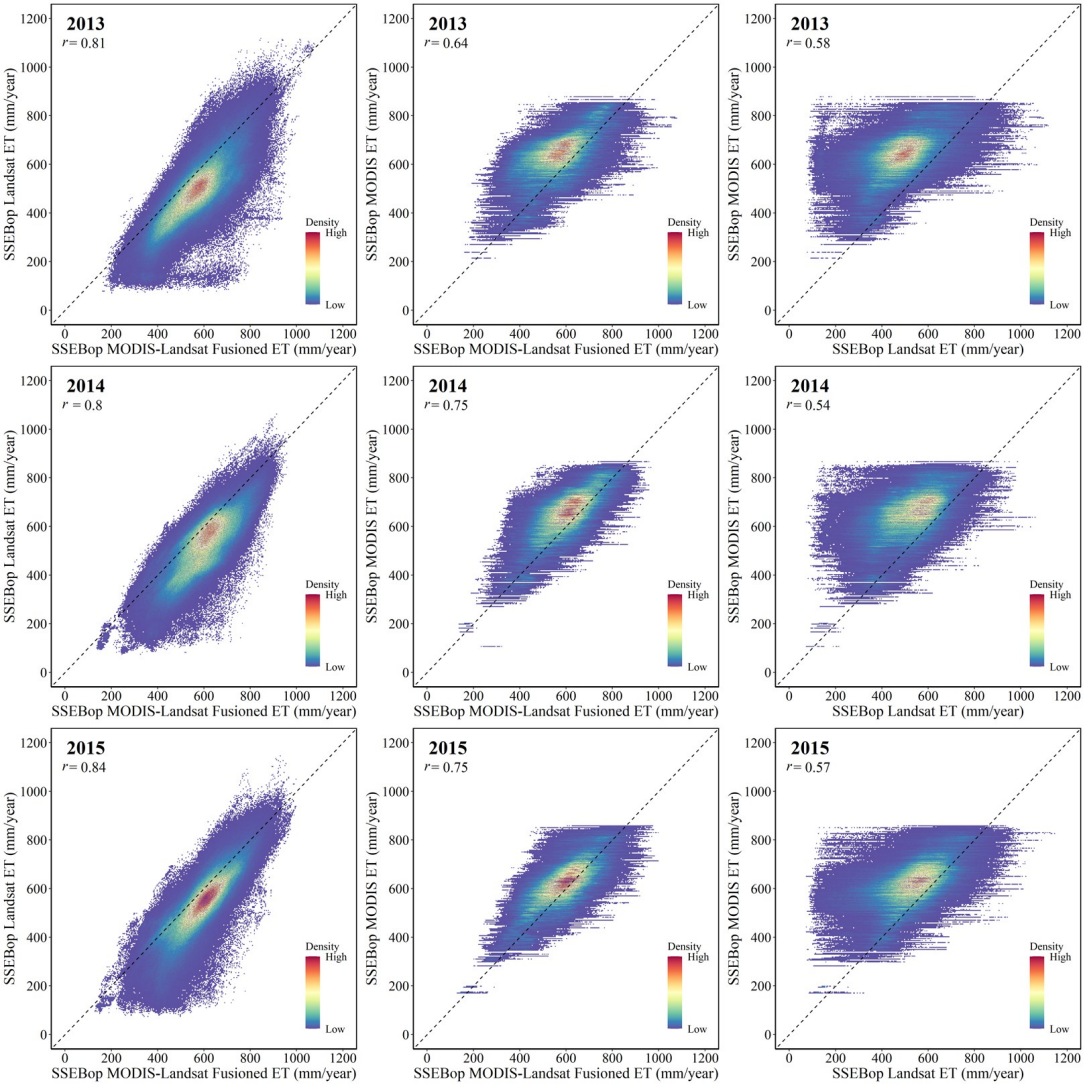
Cropland ET density scatter plots of the three methods.

We resampled the SSEBop MODIS ET to the same size as Landsat ET and plotted the cropland pixels’ ET density scatter maps of the three methods (Fig 8). Fig 8 shows that the correlation coefficient (*r*) between MODIS-Landsat Fusioned ET and SSEBop Landsat ET was the highest. The correlation between SSEBop MODIS and SSEBop MODIS-Landsat Fusioned ET was higher than that between SSEBop MODIS and SSEBop Landsat ET. The red part of the density scatters diagram is the concentrated distribution area of the central ET values. Through comparison, it showed that in the whole region, the farmland yearly ET of SSEBop MODIS was concentrated in 600–800 mm, while that of SSEBop MODIS-Landsat Fusioned ET was focused on 500–700 mm, and that of SSEBop Landsat was the lowest, concentrated in 400–600 mm. In terms of site scale validation, SSEBop MODIS can effectively reflect the subtle temporal changes of farmland ET due to its higher time resolution surface temperature. By comparing ET spatial distribution, we can conclude that SSEBop MODIS-Landsat Fusioned ET also had higher accuracy in spatial distribution because it was closer to the results of SSEBop MODIS.

**Fig 9:**
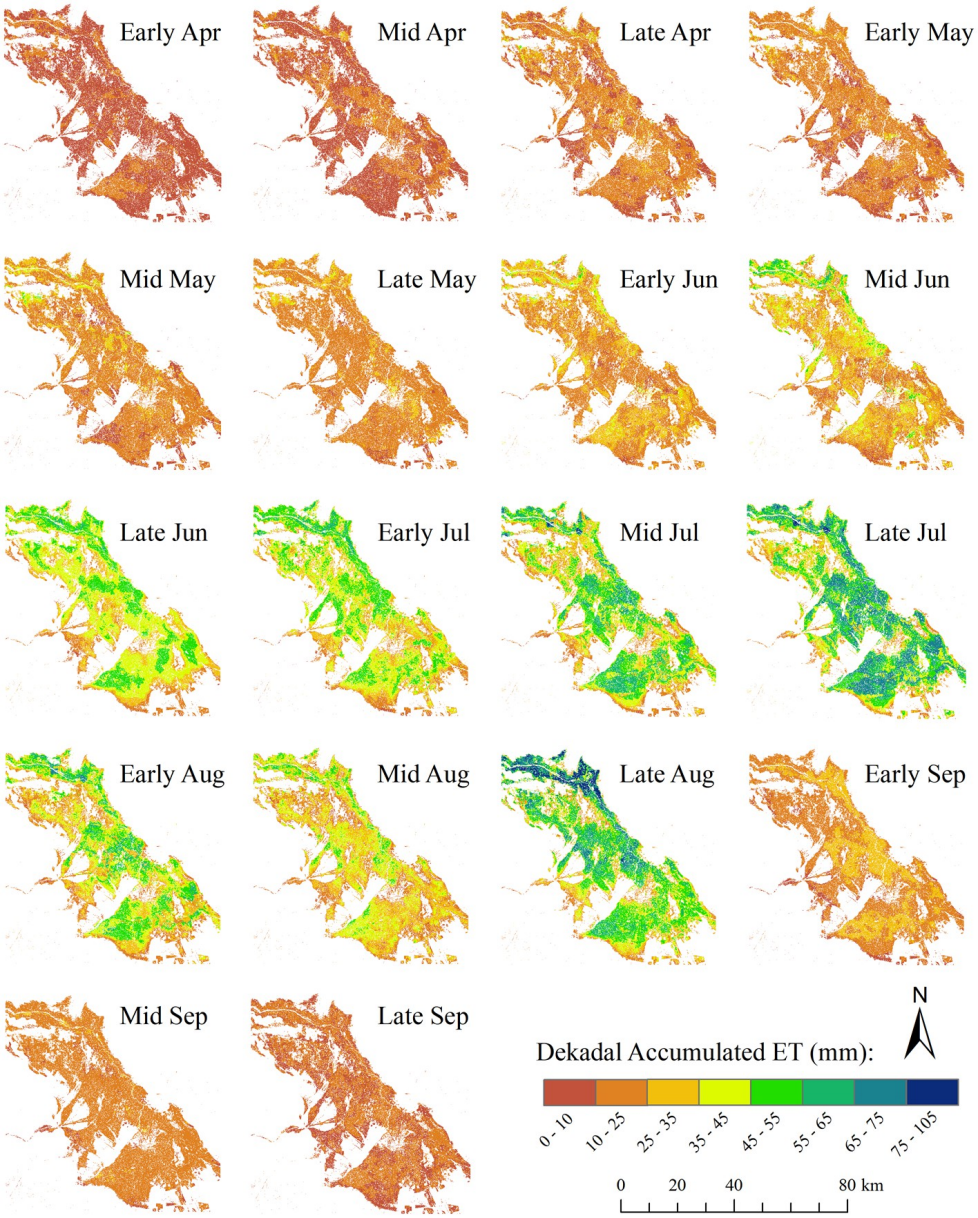
The map of dekadal accumulated SSEBop MODIS-Landsat Fusioned ET in 2015.

SSEBop MODIS-Landsat Fusioned ET can provide daily field water consumption, which provides a basis for dekadal (10 days) cumulative ET mapping. Fig 9 shows the map of dekadal accumulated SSEBop MODIS-Landsat Fusioned ET in 2015. Under the same ET mapping scale, the dekadal ET map reflected ET’s apparent change in crop growth. The cropland ET in this area was more significant from July to August, peaked in late July, and gradually decreased. It can be found from the Fig 9 that the cropland ET in late August was higher than that in mid-August. Comparing the precipitation data in Fig 5 indicates that the precipitation in late August just showed a small peak, and the sufficient water supply made the ET larger accordingly. It also confirms the effective response of surface water change to remote sensing surface temperature, which was reflected in the SSEBop ET results.

## 4. Discussion

The SSEBop model has been validated in various underlying surfaces, and the best performance was observed for croplands in recent researches [30]. Because of its simplicity and operability, it can be applied from field to continental scales. As a simplified surface energy balance model, SSEBop integrates the surface temperature, which characterizes the surface water status, and the vegetation index, which indicates the growth of crops, into the evapotranspiration fraction (*ET*_*f*_). After obtaining the *ET*_*f*_ under clear-sky conditions, SSEBop expands the *ET*_*f*_ on cloudy or non-satellite overpass days through time linear interpolation to reconstruct the ET time series. The *ET*_*f*_ can be considered as one of the most key inversion parameters in the SSEBop model. The reference evapotranspiration (ETr) usually expresses the ET potential under current meteorological conditions. Thus, the *ET*_*f*_ is the only parameter that can characterize the ET heterogeneity of different underlying surfaces.

At present, the daily field-scale evapotranspiration estimation using SSEBop and Landsat still faces uncertainty. The main reason is the lack of sufficient effective *ET*_*f*_ to describe the change of surface vegetation and water conditions. Affected by the revisit period and weather factors, less than 10 Landsat images can be obtained in a year, which has significant limitations on the simulation of severe evapotranspiration changes in arid areas of China. Although the spatial resolution of the MODIS sensor is coarser, it can revisit every day and obtain the information of surface temperature and vegetation index under clear-sky conditions.

It effectively improves earth observation data’s temporal and spatial resolution by MODIS and Landsat data fusion. However, this method has not been applied to the SSEBop model. The validation of this paper suggests that the simulation accuracy of field-scale daily evapotranspiration can be significantly improved by fusing MODIS and Landsat ET_f_ computed by SSEBop under clear-sky conditions. In many previous studies, daily high-resolution evapotranspiration estimation is often carried out by fusing surface temperature and surface reflectance to improve model input parameters’ temporal and spatial resolution. However, the spatio-temporal fusion algorithm has errors, and the errors caused by more fusion times will eventually be propagated into the output of the ET model. The spatio-temporal fusion needs to consider the contribution of effective pixels around the predicted pixels, and it is usually assumed that there is no obvious land use change between the predicted image and the images used for fusion on clear days. Therefore, we can choose the data completely free from cloud pollution for fusion as far as possible, and combine more available remote sensing images to avoid the error caused by the large time difference between the images used for fusion on clear days and predicted images. We also did the land surface temperature and vegetation fusion to test the SSEBop model, but worse results were found than the *ET*_*f*_ fusion-based method proposed in this study (not shown).

Some other model uncertainties can be found in this study, such as the systematic underestimation of ET by the three methods. In the traditional crop coefficient approach for ET estimation in irrigated agricultural areas, the product of *k*_*max*_ and *ET*_*f*_ is equivalent to crop coefficient. In a recent study, *k*_*max*_ was taken as the maximum crop coefficient value if it was available in the look-up table, which was calibrated by field data [30, 31]. Therefore, *k*_*max*_ is also a very sensitive parameter. For example, its 10% estimation error can lead to a 10% error of evapotranspiration. Therefore, such systematic underestimation errors may come from the reference evapotranspiration estimated by ERA5 reanalysis data and the *k*_*max*_ value that has not been locally corrected by EC observations or soil water balance methods.

Field-scale daily evapotranspiration mapping provides more possibilities for precision irrigation management. In the future application research of the SSEBop model, we can pay more attention to the calibration of key parameters of different crop types. At the same time, we can compare and analyze the impact of different meteorological data products, such as ERA5, CMADS, and GLDAS data [32, 33] on the performance of the model; or add more thermal infrared remote sensing data, such as sentinel-3A product [34], to improve data fusion accuracy and the performance of the SSEBop ET.

## 5. Conclusions

The *ET*_*f*_ is a key inversion parameter in the SSEBop model, which contains comprehensive information on underlying surface temperature (moisture information) and vegetation index (crop growth). In this paper, the *ET*_*f*_ values calculated by SSEBop with MODIS and Landsat were spatio-temporal fusioned to obtain the Landsat scale clear days’ *ET*_*f*_, which was further used to estimate the daily ET at the field scale.

The validation at the site scale showed that the SSEBop MODIS had the best estimation accuracy, while SSEBop with only Landsat had the worst result. The estimation accuracy of daily high-resolution ET can be effectively improved by integrating MODIS and Landsat *ET*_*f*_. Firstly, the results from the fusion scheme’s statistical parameters were better than the original scheme, with higher NSE values, lower RMSE, and PBias values. Secondly, the fusion scheme can significantly improve the underestimation of crop ET in the research area in March by the SSEBop Landsat method. The increase of ET in this period is caused by irrigation before sowing. It is difficult to capture the change of surface temperature at this time only using limited Landsat data. Finally, this fusion scheme can reduce the proportion of deviation and increase the proportion of random error in the total errors of SSEBop daily ET. The random error can be reduced with time and space, and the estimation accuracy in long-time series ET estimation will improve.

In future research, multi-source remote sensing data, such as the MODIS Aqua, ASTER, and Sentinel data, can be introduced to obtain more effective *ET*_*f*_ values to improve spatio-temporal accuracy. The SSEBop model parameters can be calibrated in combination with ground data to further improve the estimation accuracy of the model.
